# Total sleep deprivation alters spontaneous brain activity in medical staff during routine clinical work: a resting-state functional MR imaging study

**DOI:** 10.3389/fnins.2024.1377094

**Published:** 2024-04-04

**Authors:** Cong Peng, Dingbo Guo, Liuheng Liu, Dongling Xiao, Lisha Nie, Huilou Liang, Dajing Guo, Hua Yang

**Affiliations:** ^1^The Department of Radiology, Chongqing Hospital of Traditional Chinese Medicine, Chongqing, China; ^2^Department of Radiology, Second Affiliated Hospital of Chongqing Medical University, Chongqing, China; ^3^Department of Radiology, Chongqing General Hospital, Chongqing, China; ^4^Department of Anatomy, Key Lab for Biomechanics and Tissue Engineering of Chongqing, Army Medical University (Third Military Medical University), Chongqing, China; ^5^GE Healthcare, MR Research, Beijing, China

**Keywords:** total sleep deprivation, resting-state functional MRI, routine clinical, amplitude of low-frequency fluctuation (ALFF), regional homogeneity (ReHo), functional connectivity

## Abstract

**Objectives:**

To assess the effect of total sleep deprivation (TSD) on spontaneous brain activity in medical staff during routine clinical practice.

**Methods:**

A total of 36 medical staff members underwent resting-state functional MRI (rs-fMRI) scans and neuropsychological tests twice, corresponding to rested wakefulness (RW) after normal sleep and 24 h of acute TSD. The rs-fMRI features, including the mean fractional amplitude of low-frequency fluctuation (mfALFF), z-score transformed regional homogeneity (zReHo), and functional connectivity (zFC), were compared between RW and TSD. Correlation coefficients between the change in altered rs-fMRI features and the change in altered scores of neuropsychological tests after TSD were calculated. Receiver operating characteristic (ROC) and logistic regression analyses were performed to evaluate the diagnostic efficacy of significantly altered rs-fMRI features in distinguishing between RW and TSD states.

**Results:**

Brain regions, including right superior temporal gyrus, bilateral postcentral gyrus, left medial superior frontal gyrus, left middle temporal gyrus, right precentral gyrus, and left precuneus, showed significantly enhanced rs-fMRI features (mfALFF, zReHo, zFC) after TSD. Moreover, the changes in altered rs-fMRI features of the right superior temporal gyrus, bilateral postcentral gyrus, left middle temporal gyrus, and left precuneus were significantly correlated with the changes in several altered scores of neuropsychological tests. The combination of mfALFF (bilateral postcentral gyrus) and zFC (left medial superior frontal gyrus and left precuneus) showed the highest area under the curve (0.870) in distinguishing RW from TSD.

**Conclusion:**

Spontaneous brain activity alterations occurred after TSD in routine clinical practice, which might explain the reduced performances of these participants in neurocognitive tests after TSD. These alterations might be potential imaging biomarkers for assessing the impact of TSD and distinguishing between RW and TSD states.

## Introduction

Lack of sleep has become a worldwide health problem, and medical staff members are no exception (Basner et al., 2013). In fact, due to the increasing demand for medical treatment and various medical emergencies, medical staff are under higher pressure, as they usually need to work with mental fatigue and in a state of sleep deprivation (Basner et al., 2013).

Sleep deprivation is a state of sleep loss induced by various reasons, which can lead to physiological and psychological changes, causing various cognitive impairments (Fullagar et al., 2015). Depending on the amount of sleep loss, sleep deprivation can be classified into two categories, i.e., total (TSD) and partial (PSD), where the former refers to the complete absence of sleep for at least 24 h (Reynolds and Banks, 2010). Sleep deficit can significantly impact various cognitive functions, including attention, working memory, short-term memory, hippocampus-dependent memory, processing speed, and reasoning (Lim and Dinges, 2010; Krause et al., 2017). Moreover, it may jeopardize the health of medical staff and reduce the quality of medical treatment (Johnson et al., 2014; Neville et al., 2017; Yan et al., 2023). Besides, sleep deprivation causes dysregulation of sleep–wake cycle and the neuroprotective effect of sleep deprivation for short periods may participant in the sleep–wake cycle alterations (Zhou, 2019).

TSD can lead to cognitive impairments, and there are still numerous questions to be answered about the neural changes underlying these impairments (Krause et al., 2017). Functional MRI-based neuroimaging provides a noninvasive *in vivo* way to explore the mechanism. Previous studies have revealed alterations in brain activity in subjects after TSD under controlled laboratory conditions (Chee and Choo, 2004). Resting-state functional MRI (rs-fMRI) is a brain functional imaging technique that measures blood oxygen level-dependent (BOLD) signals when subjects are in the resting state (Biswal et al., 1995; Lv et al., 2018). Deantoni et al. utilized rs-fMRI to investigate sleep-related alterations in seed-based functional connectivity (FC) and amplitude of low-frequency fluctuation (ALFF) after spatial navigation learning and relearning (Deantoni et al., 2021). Network changes were also observed through rs-fMRI-based neuroimaging. A previous study proved that TSD reduces the network modularity of the limbic, default mode, salience, and executive modules, and these changes were associated with the behavioral impairments elicited by TSD (Ben Simon et al., 2017). Therefore, rs-fMRI can serve as a promising imaging technique for exploring the underlying mechanism of how TSD impacts cognition.

Most previous TSD studies were performed under controlled laboratory conditions, where subjects were usually instructed to engage in low-intensity activities during TSD, such as reading, talking to their partners, or watching movies (Lei et al., 2015). In contrast, in routine clinical practice, medical staff must work in a fast-paced and high-pressure environment during their night shifts, which is markedly different from experimental TSD conditions (Curtis et al., 2019). Therefore, the effects of TSD on the brain are likely to be diverse under different conditions (Eide et al., 2021). Given that medical work is a matter of life and death, it is important to investigate the potential harm of TSD to medical staff. Besides, there may be some beneficial effects of education on brain neuroplasticity and neuroprotection, considering the relatively higher education levels in medical doctors and nurses (Zhou, 2023).

This study aimed to assess the effects of TSD on the brain functioning of medical staff under routine clinical conditions. After experiencing TSD, all neurobehavioral functions returned to baseline levels after one recovery night, except self-reported vigor (Yamazaki et al., 2021). We compared the alterations in spontaneous brain activity and performance on neuropsychological tests between rested wakefulness (RW) after normal sleep and TSD states (at least 2 weeks apart). Given that ALFF and regional homogeneity (zReHo) are indicators of brain activity (Luo et al., 2016), while FC usually reorganized to compensate for any possible local neuronal injuries (Sheth et al., 2021), we analyzed spontaneous brain activity patterns in medical staff using rs-fMRI with mean fractional ALFF (mfALFF), z-score transformed regional homogeneity (zReHo), and z-score transformed FC (zFC) algorithms. The possible associations between these brain activity patterns and scores of neuropsychological tests were also investigated to seek potential clinical imaging biomarkers for assessing the impact of TSD. We hypothesized that TSD would affect medical staff’s resting-state spontaneous brain activity and their performance on neuropsychological tests and that alterations in brain activity patterns might serve as imaging biomarkers to distinguish between RW and TSD brain states.

## Methods

### Participants

A total of 39 healthy adult subjects (17 females) aged from 26 to 42 years (mean age = 31.85 ± 4.36 years) participated in this study. They were all medical staff (doctors and nurses) from our hospital, who rotated through night shifts on a regular schedule (2–4 night shifts per month), the remaining days were for normal sleep. All subjects met the following inclusion criteria: (1) no history of neurological or psychiatric disorders; (2) absence of sleep disorders; (3) no history of smoking, alcoholism, or drug abuse; (4) absence of claustrophobia; (5) normal sleep habits (more than 6.5 h of sleep at night; going to bed no later than 1:00 AM; and waking up no later than 9:00 AM) (Chee et al., 2011) when not on night shift duty. The studies involving human participants were reviewed and approved by Ethics Committee of Chongqing Hospital of Traditional Chinese Medicine (institutional review board (IRB) approval number: 2023-KY-KS-PC). The participants provided their written informed consent to participate in this study. Three subjects were excluded from the final analyses because of excessive head movement (*n* = 1) or loss to follow-up (*n* = 2).

### Neuropsychological tests

To evaluate the impact of TSD on the cognitive functions of medical staff, neuropsychological tests, including the Number Connection Test-A (NCT-A), Digit Symbol Test (DST), Line Tracing Test (LTT), Serial Dotting Test (SDT) (Li et al., 2013), were performed in this study. These neuropsychological tests are usually used to assess various aspects of cognitive functions such as attention, processing speed, motor coordination, visual perception, visual–spatial orientation, and memory (Weissenborn et al., 2001; Strauss et al., 2006; Lezak et al., 2012). Specifically, the NCT-A assesses attention, mental flexibility, and psychomotor speed by requiring participants to connect numbers sequentially as quickly as possible. The DST measures cognitive processing speed, attention, and working memory by asking participants to match symbols with corresponding numbers within a given time limit. The LTT evaluates visuospatial abilities and motor coordination by instructing participants to trace lines accurately and swiftly. Lastly, the SDT measures fine motor skills and hand-eye coordination by having participants connect a series of dots in a specific order.

The DST results were measured in points, while the NCT-A and SDT results were measured in seconds, including the time required to correct errors. The LTT results were measured both in terms of the time required to complete the test (LTTt, seconds) and the error score (LTTe), with LTT = (1 + LTTe/100) × LTTt (Amodio et al., 2008; Li et al., 2013). Therefore, a higher DST score indicated better performance, while lower scores on the other tests indicated better performance.

### Study protocol

All subjects were interviewed three times during the study. During the first visit, the protocol and purpose of the study were briefly introduced. All subjects consented to undergo two MRI scans during the last two visits at 7:30 a.m., which were at least 2 weeks apart. One scan was conducted during RW after a normal night’s sleep, while the other was performed after TSD. Neuropsychological tests followed both MRI scans. All subjects declared no consumption of stimulants or caffeine (coffee, tea, cola, etc.) for at least 24 h before the study. The TSD process started at 7:30 a.m. on the first day and ended at 7:30 a.m. on the next day. During this period, the subjects were instructed to stay awake for 24 h by participating in their routine clinical night shift duty and engaging in some low-intensity work during non-night shift hours. To guarantee their alertness throughout the TSD period, we employed a two-pronged verification process. Firstly, we conducted a thorough examination of the monitoring systems at their respective work sites, the majority of which were equipped with surveillance cameras. Secondly, subjects were instructed to self-report any instances of falling asleep simultaneously. To reinforce this, we cross-validated the gathered data by scrutinizing the information system employed by subjects for patient intake. This system meticulously recorded the time and number of patients seen, providing an additional layer of confirmation that participants did not experience lapses into sleep during the study. In the RW session, the subjects were instructed to stay awake with daily work from 7:30 a.m. to 11:30 p.m. during the first day, followed by a scheduled sleep period from 11:30 p.m. to 7:30 a.m. on the second day.

The subjects were equally and randomly divided into two groups and trained before tests to reduce the influence of practice effects and novelty on the MRI environment. Half of the subjects began with the RW session, while the other half commenced with the TSD session.

### Functional MRI acquisition

Resting-state fMRI data acquisition was performed on a 3.0 T MRI scanner (SIGNA Architect, GE Healthcare, USA) equipped with a 48-channel head coil. The gradient echo echo-planar imaging (EPI) pulse sequence was used to acquire BOLD functional images with the following parameters: repetition time (TR) = 2000 ms, echo time (TE) = 30 ms, flip angle (FA) = 90°, 34 slices with interleaved acquisition, no gap, slice thickness = 5 mm, field of view (FOV) = 220 mm × 220 mm, acquisition matrix = 64 × 64, and a total of 240 volumes. During the scan, these subjects were instructed to stay awake and focus on the fixation point to significantly increase the likelihood of the subjects remaining stably awake (Tagliazucchi and Laufs, 2014) and remain as still as possible while their head was properly restrained to minimize involuntary movement effects (Peng et al., 2021).

### Image pre-processing

Data Processing and Analysis for Brain Imaging (DPABI) (Yan et al., 2016) based on the statistical parameter mapping software package (SPM12)^1^ were used to preprocess the original fMRI data in MATLAB (The MathWorks Inc., Natick, MA, United States). Firstly, the initial 10 volumes of the image data were eliminated to exclude the influence caused by the inhomogeneity of the initial magnetic field. Then, after correcting the difference in acquisition time, the processing steps of the remaining 230 volumes were as follows: slice-timing and head motion correction, spatial normalization (voxel size = 3 mm × 3 mm × 3 mm), Gaussian smoothing with 4 mm full width at half maximum (FWHM), regression to remove covariates (head motion parameters, cerebrospinal fluid signals, white matter signals, global mean signals), linear drift and low-frequency filtering (0.01–0.08 Hz). Next, rs-fMRI features, including mfALFF, zReHo, and zFC, were calculated.

### Calculation of mfALFF, zReHo, and zFC

The mfALFF directly reflects the intensity of spontaneous brain activities (Luo et al., 2016; Ren et al., 2016). The time series for each voxel was transformed to the frequency domain by a fast Fourier transform to obtain the power spectrum. Next, the square root of the power spectrum was calculated at each frequency. The fALFF is the ratio of the sum of amplitudes at the low-frequency range (0.01–0.08 Hz) to that of the entire frequency range, which has been shown to be less susceptible to physiological noise than ALFF (Zou et al., 2008). For normalization, the fALFF value of each voxel was divided by the whole-brain average fALFF to obtain the mean fALFF (mfALFF) (Yu et al., 2021).

ReHo reflects the local synchronization of spontaneous neural activities between neighboring voxels and is commonly used to investigate the local functional connectivity of brain regions (Fu et al., 2018; Lin et al., 2018; Peng et al., 2021). Kendall’s coefficient of concordance (KCC) was calculated to measure the local synchronization of a given voxel to its 26 nearest neighboring voxels, i.e., the ReHo value of this voxel (Zang et al., 2004). Then, the ReHo value was spatially smoothed (FWHM = 6 mm) and normalized by z-score transformation to obtain the zReHo.

Finally, seed-based FC was applied to measure the temporal synchronization of the spontaneous brain activity among different brain regions (Qiu et al., 2014) and thus reflect brain intrinsic functional organization (Joel et al., 2011). By defining brain regions with significant changes in mfALFF or zReHo as regions of interest (ROIs), we used seed-based FC to detect the potential brain function alterations in subjects after TSD (Zhang et al., 2023). FC was also normalized by z-score transformation to obtain the zFC.

### Statistical analysis

All statistical analyses were performed using IBM SPSS Statistics for Windows (Armonk, NY: IBM Corp., version 22.0) and MedCalc Statistical Software (Ostend, Belgium: MedCalc Software Ltd., version 18.2). A *p* < 0.05 was considered statistically significant. Data were presented as mean ± standard deviation for continuous variables and number (percentage) for categorical variables.

The normality of the variable distribution was assessed using the Shapiro–Wilk normality test. For continuous variables with a normal distribution, the paired samples t-test was used to compare the neuropsychological test scores between TSD and RW states. The Wilcoxon signed-rank test was employed for continuous variables with a non-normal distribution. The one-sample t-test was conducted to assess the group mean of mfALFF, zReHo, and zFC maps. The paired samples t-test was also used to investigate the differences in mfALFF, zReHo, and zFC in regional brain areas between TSD and RW states, with mean head motion parameters included as covariates. The resulting statistical maps were false discovery rate (FDR) or family-wise error (FWE) corrected. The Spearman correlation analysis was performed to investigate the correlation between the change in significantly altered rs-fMRI features (TSD - RW) and the change in significantly altered scores of neuropsychological tests (TSD – RW).

Binary logistic regression with a forward approach was performed to build individual or combined models using significantly altered rs-fMRI features and explore associations between significantly altered rs-fMRI features and brain states (TSD or RW state). Receiver operating characteristic (ROC) curve analyses with an area under the curve (AUC) were used to evaluate the diagnostic efficacy of models with single or multiple parameters in distinguishing between TSD and RW brain states. The AUC values ranging from 0.9 and 1 were considered excellent, 0.8 and 0.9 good, 0.7 and 0.8 fair, 0.6 and 0.7 poor, and < 0.6 failed (El Khouli et al., 2009). DeLong’s test was used to compare the differences between the AUCs of different ROC curves.

## Results

### Demographic characteristics and cognitive performance

The final sample included 36 healthy subjects (15 females, 31.97 ± 4.29 years). Table 1 summarizes the demographic details of the subjects and their performance on neuropsychological tests. The DST score of the subjects in the TSD state was significantly lower than that in the RW state (*t* = 4.182, *p* < 0.001). Additionally, the NCT-A score of the subjects in the TSD state was higher than that in the RW state (*z* = −2.35, *p* = 0.019). Furthermore, the LTT score of the subjects in the TSD state was significantly higher than that in the RW state (*z* = −2.29, *p* = 0.02). The SDT score of the subjects in the TSD state was also higher than that in the RW state, but the difference was not statistically significant.

**Table 1:** Demographic and cognitive performance.

Parameters	TSD (*n* = 36)	RW (*n* = 36)	*t/z* value	*p*-value
Sex (M/F)	21/15	21/15	–	–
Age (years)	31.97 ± 4.29	31.97 ± 4.29	–	–
DST	64.81 ± 8.93	69.58 ± 9.43	4.18	<0.001^a^
NCT-A (sec)	31.86 ± 8.70	29.28 ± 9.56	−2.35	0.019^b^
LTT (sec)	54.69 ± 23.76	49.06 ± 17.32	−2.29	0.02^b^
SDT (sec)	42.44 ± 8.54	41.33 ± 9.89	−1.71	0.087^b^

TSD, total sleep deprivation for 24 h; RW, rested wakefulness after normal sleep; DST: Digit Symbol Test; NCT-A: Number Connection Test-A; LTT: Line Tracing Test; SDT: Serial Dotting Test. ^a^ Paired samples t-test, ^b^ Wilcoxon signed-rank test.

### Changes in rs-fMRI feature between RW and TSD states

#### mfALFF changes between RW and TSD states

**Figure 1:**
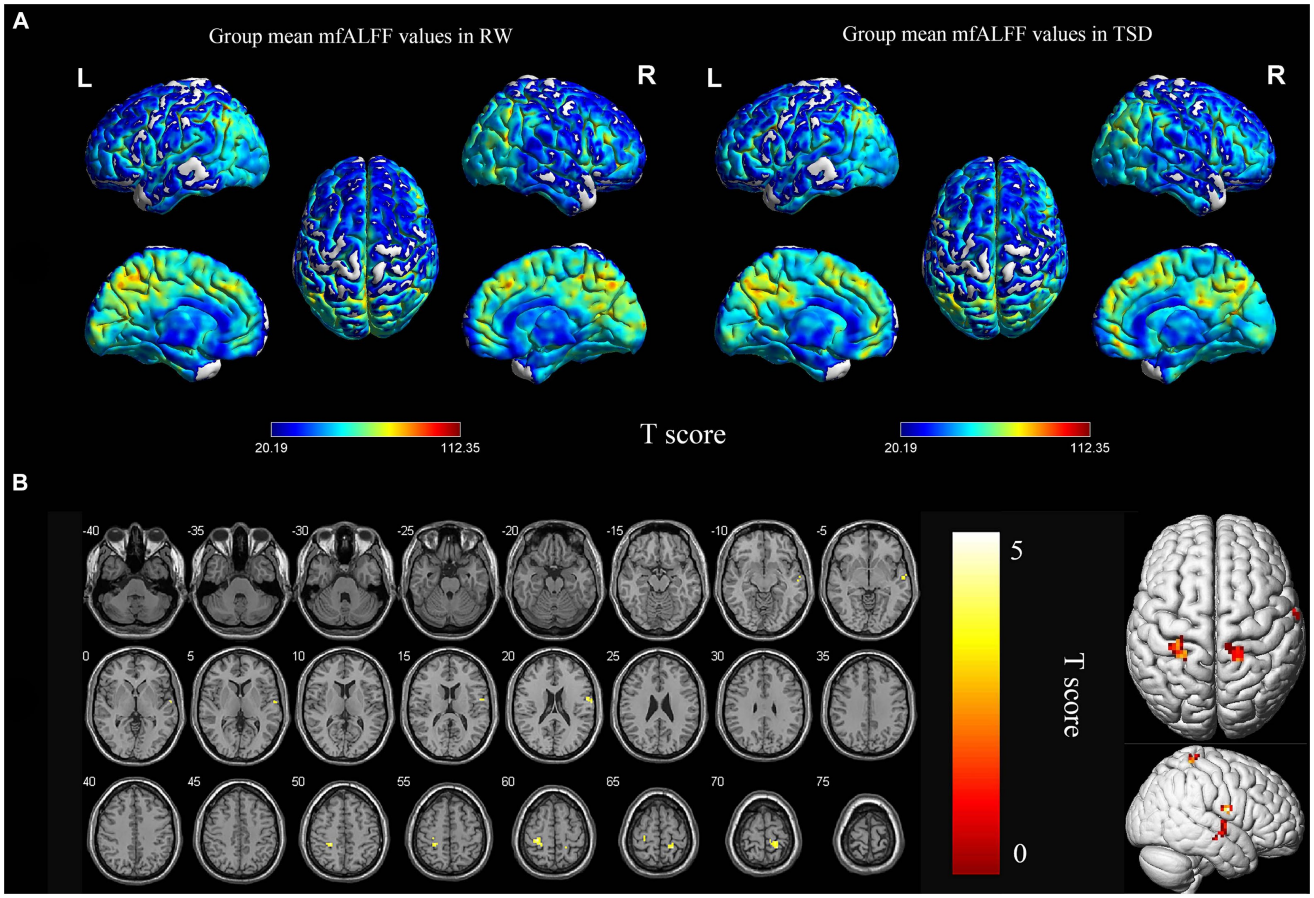
Spatial maps of within-group mean mfALFF in RW and TSD **(A)**, and mfALFF differences between RW and TSD **(B)**. Right superior temporal gyrus, bilateral postcentral gyrus showed significantly enhanced (yellow) mfALFF after TSD **(B)**. The range of the T score is represented by the color bar (*p* < 0.05, FDR corrected at the cluster level, initial voxel-wise threshold *p* = 0.001). mfALFF, mean fractional amplitude of low-frequency fluctuation; RW, rested wakefulness; TSD, total sleep deprivation.

According to the analysis of mfALFF, the spontaneous brain activity in the right superior temporal gyrus and bilateral postcentral gyrus were significantly enhanced after TSD (*p* < 0.05, FDR corrected at the cluster level, initial voxel-wise threshold *p* = 0.001) (Table 2; Figure 1).

**Table 2:** Regions showing mfALFF and zReHo differences between TSD and RW.

	Brain regions	Side	AAL	Voxels with the maximum effect				
				MNI (x, y, z)			voxels	*t* value
mfALFF	Superior temporal gyrus	R	82	60	−6	−3	15	4.4184
	Postcentral gyrus	R	58	54	−3	18	15	4.2296
	Postcentral gyrus	L	57	−27	−36	57	33	4.9971
zReHo	Superior temporal gyrus	R	82	39	−18	15	81	4.8512
	Postcentral gyrus	R	58	21	−39	63	185	5.2551
	Postcentral gyrus	L	57	−15	−37	62	11	5.2551

Paired samples *t*-test was used to compare the differences between TSD and RW. AAL, Automated Anatomical Labeling atlas’s area; MNI, Montreal Neurologic Institute.

#### zReHo changes between RW and TSD states

**Figure 2:**
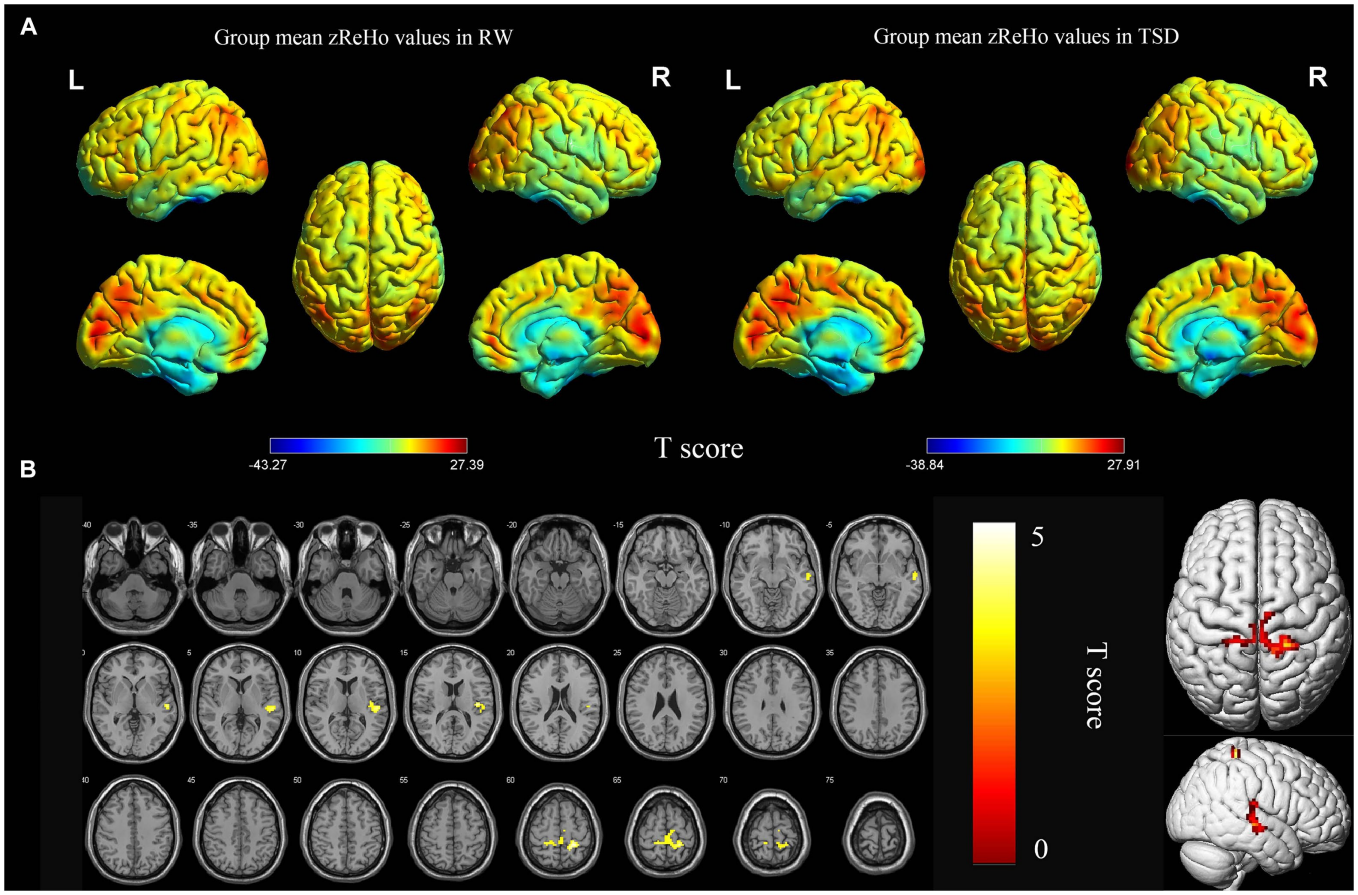
Spatial maps of within-group mean zReHo in RW and TSD **(A)**, and zReHo differences between RW and TSD **(B)**. Right superior temporal gyrus, bilateral postcentral gyrus showed significantly enhanced (yellow) zReHo after TSD **(B)**. The range of the T score is represented by the color bar (*p* < 0.05, FDR corrected at the cluster level, initial voxel-wise threshold *p* = 0.001). zReho, z-score transformed regional homogeneity; RW, rested wakefulness; TSD, total sleep deprivation.

According to the analysis of zReHo, the local functional connectivity in the right superior temporal gyrus and bilateral postcentral gyrus significantly increased after TSD (*p* < 0.05, FDR corrected at the cluster level, initial voxel-wise threshold *p* = 0.001) (Table 2; Figure 2).

#### zFC changes between RW and TSD states

**Figure 3:**
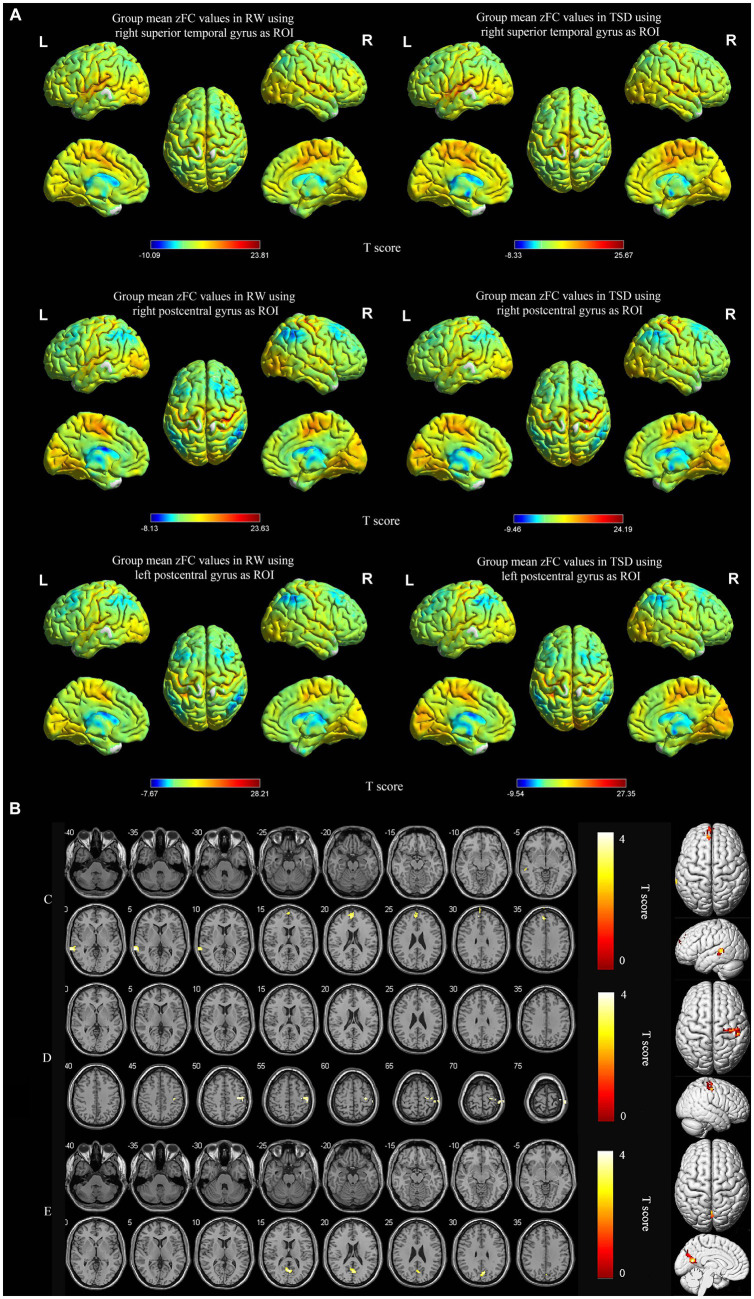
Spatial maps of within-group mean zFC in RW and TSD **(A)**, and zFC differences between RW and TSD **(B)**. zFC between right superior temporal gyrus and left medial superior frontal gyrus, left middle temporal gyrus enhanced after TSD **(C)**. zFC between right postcentral gyrus and right precentral gyrus enhanced after TSD **(D)**. zFC between left postcentral gyrus and left precuneus gyrus enhanced after TSD **(E)**. The color bar represents T score (*p* < 0.05, FWE corrected at the cluster level, initial voxel-wise threshold *p* = 0.001). Yellow indicates positive and enhanced functional connectivity. zFC, z-score transformed functional connectivity; RW, rested wakefulness; TSD, total sleep deprivation.

Using the right superior temporal gyrus as the seed point, its functional connectivity with the left medial superior frontal gyrus and left middle temporal gyrus was significantly enhanced after TSD (*p* < 0.05, FWE corrected at the cluster level, initial voxel-wise threshold *p* = 0.001) (Table 3; Figure 3A). Using the right postcentral gyrus as the seed point, its functional connectivity with the right precentral gyrus was significantly enhanced after TSD (*p* < 0.05, FWE corrected at the cluster level, initial voxel-wise threshold *p* = 0.001) (Table 3; Figure 3B). Using the left postcentral gyrus as the seed point, its functional connectivity with the left precuneus was significantly enhanced after TSD (*p*<0.05, FWE corrected at the cluster level, initial voxel-wise threshold *p* = 0.001) (Table 3; Figure 3C).

**Table 3:** Regions showing zFC differences between TSD and RW.

ROIs	Brain regions	Side	AAL	Voxels with the maximum effect				
				MNI (x, y, z)			voxels	*t* value
ROI1	Medial superior frontal gyrus	L	23	−6	45	39	89	4.0238
ROI1	Middle temporal gyrus	L	85	−75	−36	3	55	4.6240
ROI2	Precentral gyrus	R	2	33	−21	63	46	4.0918
ROI3	Precuneus	L	67	0	−66	18	70	4.8005

Paired samples *t*-test was used to compare the differences between TSD and RW. ROI1, right superior temporal gyrus; ROI2, right postcentral gyrus; ROI3, left postcentral gyrus.

### Spearman correlation analysis between the change in altered rs-fMRI features and the change in altered scores of neuropsychological tests after TSD

**Figure 4:**
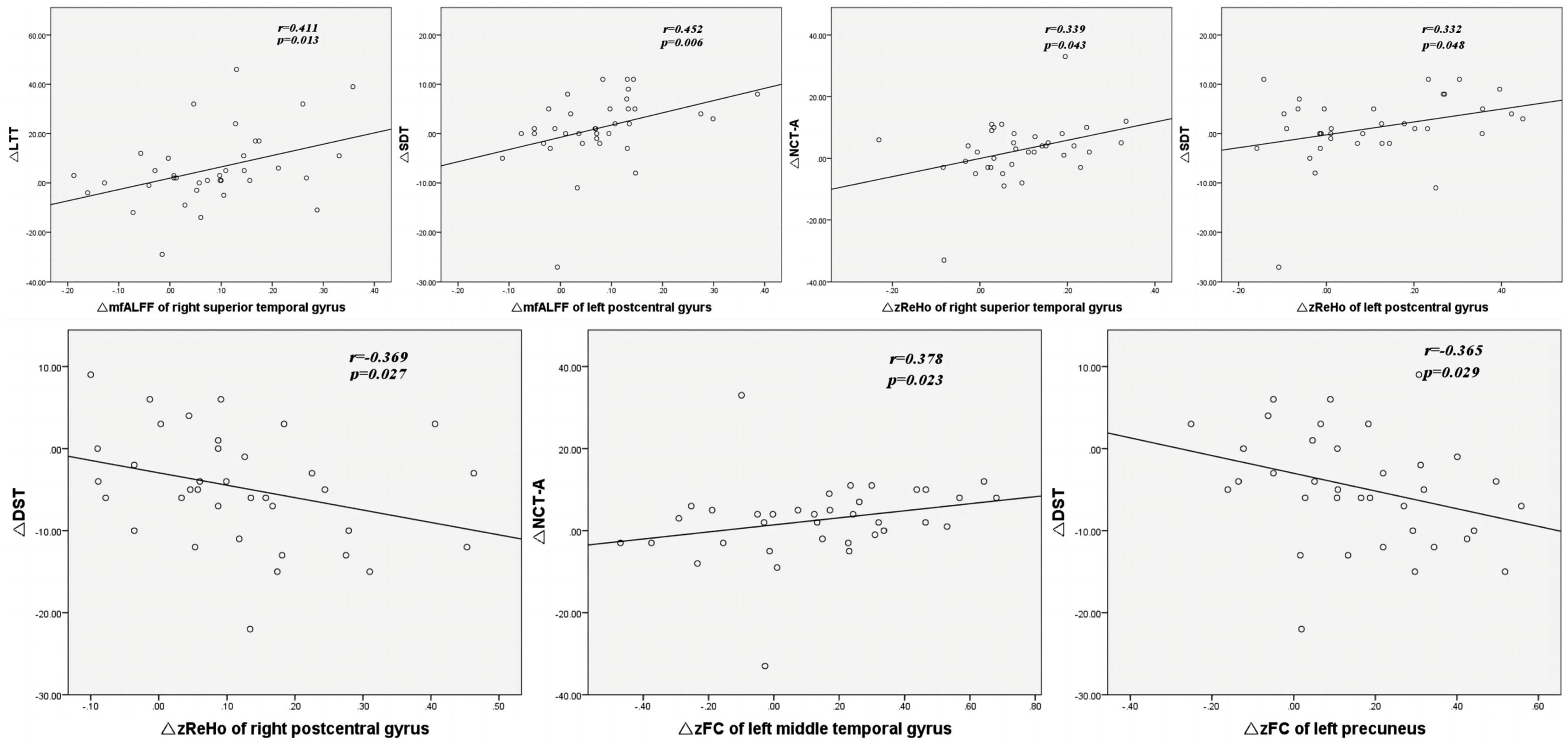
The correlation analysis between the change in mfALFF, zReHo, zFC and the change in scores of neuropsychological tests. ΔmfALFF, ΔzReho, ΔzFC, ΔNCT-A, ΔLTT, ΔDST, ΔSDT represent the change in mfALFF, zReho, zFC, NCT-A scores, LTT scores, DST scores, and SDT scores between RW and TSD states, respectively. mfALFF, mean fractional amplitude of low-frequency fluctuation; zReho, z-score transformed regional homogeneity; zFC, z-score transformed functional connectivity; DST, Digit Symbol Test; NCT-A, Number Connection Test-A; LTT, Line Tracing Test; SDT, Serial Dotting Test; TSD, total sleep deprivation.

As shown in Table 4 and Figure 4, Spearman correlation analysis revealed significant correlations between the change in altered rs-fMRI features in several brain regions and the change in altered cognitive scores after TSD. Specifically, the change in mfALFF value of the right superior temporal gyrus was positively correlated with the change in scores of LTT (*r* = 0.411, *p* = 0.013). The change in mfALFF value of left postcentral gyrus was positively correlated with the change in scores of SDT (*r* = 0.452, *p* = 0.006). The change in zReHo value of the right superior temporal gyrus exhibited a positive correlation with the change in scores of NCT-A (*r* = 0.339, *p* = 0.043). The change in zReHo value of the left postcentral gyrus exhibited a positive correlation with the change in scores of SDT (*r* = 0.332, *p* = 0.048). The change in zReHo value of the right postcentral gyrus exhibited a negative correlation with the change in scores of DST (*r* = −0.369, *p* = 0.027). The change in zFC value of left middle temporal gyrus exhibited a positive correlation with the change in scores of NCT-A (*r* = 0.378, *p* = 0.033). The change in zFC value of left precuneus gyrus exhibited a negative correlation with the change in scores of DST (*r* = 0.378, *p* = 0.033).

**Table 4:** The correlation analysis between the change in mfALFF, zReHo, zFC and the change in scores of neuropsychological tests.

Brain regions	AAL	Neuropsychological test	*r*	*p*-value
Right superior temporal gyrus (ΔmfALFF)	82	ΔLTT	0.411	0.013
Left postcentral gyrus (ΔmfALFF)	57	ΔSDT	0.452	0.006
Right superior temporal gyrus (ΔzReHo)	82	ΔNCT-A	0.339	0.043
Left postcentral gyrus (ΔzReHo)	57	ΔSDT	0.332	0.048
Right postcentral gyrus (ΔzReHo)	58	ΔDST	−0.369	0.027
Left middle temporal gyrus (ΔzFC)	85	ΔNCT-A	0.378	0.023
Left precuneus (ΔzFC)	61	ΔDST	−0.365	0.029

Correlation analysis was assessed by Spearman correlation. ΔmfALFF, ΔzReho, ΔzFC, ΔNCT-A, ΔLTT, ΔDST, ΔSDT represent the change in mfALFF, zReho, zFC, NCT-A scores, LTT scores, DST scores, and SDT scores between RW and TSD states, respectively. mfALFF, mean fractional amplitude of low-frequency fluctuation; zReho, z-score transformed regional homogeneity; zFC, z-score transformed functional connectivity; DST, Digit Symbol Test; NCT-A, Number Connection Test-A; LTT, Line Tracing Test; SDT, Serial Dotting Test.

### The AUCs of single- or multi-parameter models constructed by altered rs-fMRI features

**Figure 5:**
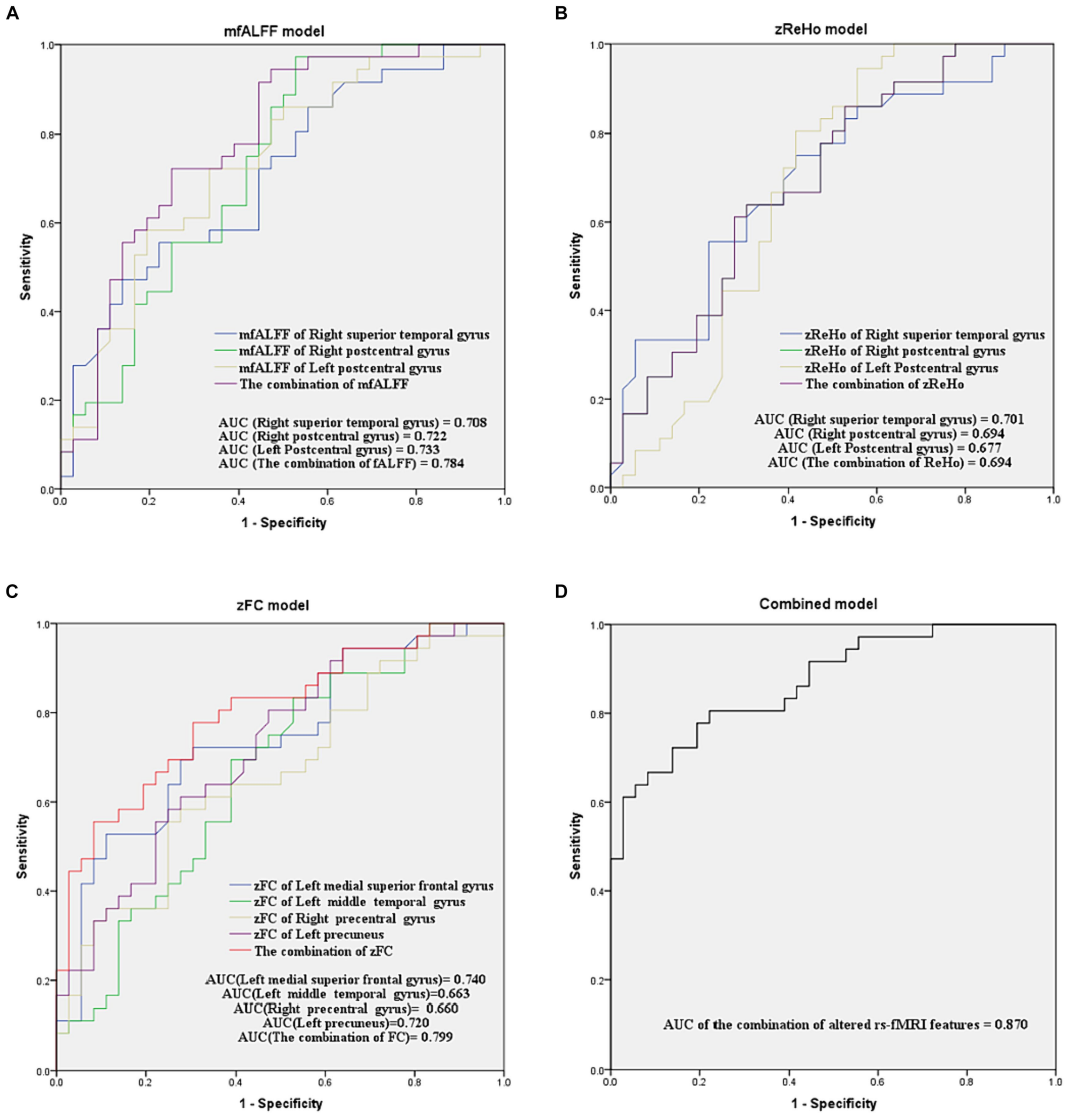
ROC curve analyses for evaluating the diagnostic efficacy of models with single or multiple parameters in distinguishing between TSD and RW brain states. **(A–D)** are models of mfALFF, zReHo, zFC, and the combination of altered rs-fMRI features, respectively. mfALFF, mean fractional amplitude of low-frequency fluctuation; zReho, z-score transformed regional homogeneity; zFC, z-score transformed functional connectivity.

As shown in Figure 5, the AUCs of single-parameter models constructed by the mfALFF value in the right superior temporal gyrus, right postcentral gyrus, and left postcentral gyrus were 0.708, 0.722, and 0.733, respectively. The AUCs of single-parameter models constructed by the zReHo value in the right superior temporal gyrus, right postcentral gyrus, and left postcentral gyrus were 0.701, 0.694, and 0.677, respectively. The AUCs of single-parameter models constructed by the zFC value in the left medial superior frontal gyrus, left middle temporal gyrus, right precentral gyrus, and left precuneus were 0.740, 0.663, 0.660, and 0.720, respectively. The combination of zFC values in the left medial superior frontal gyrus and left precuneus yielded a higher AUC (0.799), while the combination of the mfALFF or zReHo values had an AUC of 0.784 or 0.694, respectively. Next, the combined model constructed by combining the mfALFF value in the bilateral postcentral gyrus and the zFC values in the left medial superior frontal gyrus and left precuneus showed the highest AUC (0.870).

DeLong’s test showed no significant difference in AUC between the combination of mfALFF and the single mfALFF. The AUC of the combination of zFC was only superior to that of the single zFC in the right precentral gyrus (*p* = 0.047). In contrast, the AUC of the combined model constructed by combining mfALFF and zFC was significantly higher than that of each single-parameter model (*p* < 0.05, Table 5).

**Table 5:** Pairwise comparison of AUCs between combined model and other models.

Combined model vs. other models	95% CI	*z* value	*p*-value
**mfALFF model**			
mfALFF of right superior temporal gyrus	0.044–0.280	2.685	*p* = 0.007*
mfALFF of right postcentral gyrus	0.034–0.262	2.541	*p* = 0.011*
mfALFF of left postcentral gyrus	0.031–0.244	2.529	*p* = 0.011*
The combination of mfALFF	−0.005-0.178	1.858	*p* = 0.063
**zReHo model**			
zReHo of right superior temporal gyrus	0.057–0.282	2.957	*p* = 0.003*
zReHo of right postcentral gyrus	0.057–0.296	2.891	*p* = 0.004*
zReHo of left Postcentral gyrus	0.065–0.322	2.947	*p* = 0.003*
The combination of zReHo	0.057–0.296	2.891	*p* = 0.004*
**zFC model**			
zFC of left medial superior frontal gyrus	0.032–0.229	2.601	*p* = 0.009*
zFC of left middle temporal gyrus	0.078–0.337	3.136	*p* = 0.002*
zFC of right precentral gyrus	0.087–0.335	3.325	*p* < 0.001*
zFC of left precuneus	0.041–0.260	2.683	*p* = 0.007*
The combination of zFC	−0.005-0.149	1.828	*p* = 0.068

DeLong’s test was used to compare the differences between AUCs of different models. The combined model represents the combination of mfALFF of bilateral postcentral gyrus and zFC of left medial superior frontal gyrus and left precuneus. The single asterisk (*) indicates *p* < 0.05; CI, Confidence Interval.

## Discussion

This study used rs-fMRI to explore the effect of TSD on spontaneous brain activity in medical staff during fast-paced and high-pressure clinical work. Our results revealed that TSD led to increased spontaneous brain activity in several brain regions of medical staff, as reflected by altered rs-fMRI features. Furthermore, neuropsychological tests indicated a decline in some cognitive functions after TSD. Additionally, the changes in some altered rs-fMRI features were found to be significantly correlated with the changes in performance of neuropsychological tests after TSD.

Subjects in this study were medical staff experienced in routine clinical practice, rotated through night shifts on a regular schedule in fast-paced and high-pressure physical and psychological states. Medical staff are usually required to focus on their patients, as distraction or carelessness may cause more errors (Johnson et al., 2014). After experiencing TSD, all neurobehavioral functions returned to baseline after one recovery night, except self-reported vigor (Yamazaki et al., 2021). We arranged the MRI scans and neuropsychological tests for both TSD and RW states into two separate night shift cycles, with at least 2 weeks apart. To the best of our knowledge, most studies on sleep deprivation were conducted in laboratory conditions (Krause et al., 2017). The major difference between the current and previous experimental studies is in the mental stress of subjects and workload during TSD. Previous research in laboratory conditions has revealed increased rs-fMRI features (ALFF, ReHo) in the postcentral and temporal gyrus after sleep deprivation (Chen et al., 2023; Li et al., 2023). In their study, Chen et al. (2023) reported an increase of ReHo in the right postcentral gyrus after sleep deprivation, which is consistent with observations in the present research. However, we also detected increased rs-fMRI features in other brain regions after sleep deprivation. On the other hand, Li et al. (2023) found decreased ALFF in the cerebellum after sleep deprivation, which is not consistent with our results. These discrepancies could potentially be attributed to factors such as variations in sample characteristics. Unlike laboratory conditions, medical staff working in routine clinical practice perform their regular duty under high workload and mental stress during TSD, which may contribute to these differences.

Our results revealed increased mfALFF and zReho in the bilateral postcentral gyrus after TSD. The increased spontaneous activity in the postcentral gyrus is related to decreased sleep quality and increased anxiety (Gao et al., 2015; Zhou et al., 2017). The postcentral gyrus is the hub of the somatosensory network (sense of touch and kinesthesia) (Fu et al., 2019; Zhou et al., 2020), which contains the primary somatosensory cortex where somatotopic information of the contralateral half of the body is gathered (Ferro et al., 2015). Also, the sensory feedback from the somatosensory network is a critical part of the execution of voluntary manipulation. The somatosensory and somatomotor networks work together to control our movements. The somatomotor network sends orders, and the somatosensory network receives feedback messages to form a closed loop (Kato and Izumiyama, 2015). Our results showed that the changes in mfALFF and zReHo of postcentral gyrus were associated with the changes in scores of SDT and DST. The SDT measures fine motor skills and hand-eye coordination, the DST measures cognitive processing speed, and working memory which can be affected by somatosensory network. Therefore, altered spontaneous activity in the somatosensory network may influence the performance of motor-related neuropsychological tests.

Increased mfALFF and zReho were also found in the right superior temporal gyrus after TSD. The superior temporal gyrus is associated with social cognition and includes auditory and language cortices (Bigler et al., 2007; Dziobek et al., 2011). Our results showed that LTT and NCT-A scores declined after TSD, and the changes in mfALFF and zReho values in the right superior temporal gyrus were positively correlated with the changes in scores of LTT and NCT-A. These results indicated that increased spontaneous brain activity in the right superior temporal gyrus may affect subjects’ performance on LTT and NCT-A, tasks linked to functions like speed, attention, and working memory. This is reasonable because the superior temporal gyrus, as a component of the salience network (Xiong et al., 2020), participates in the regulatory hub for internal and external stimuli, guiding the generation of cognitive processes, affecting attention, working memory (Menon and Uddin, 2010).

Interestingly, the local functional connectivity reflected by zReHo also increased in the bilateral postcentral gyrus and right superior temporal gyrus after TSD, consistent with the brain regions with increased mfALFF. This demonstrated that spontaneous brain activity within these areas increased and maintained a high degree of local synchronization.

Next, we found enhanced FC between the right superior temporal gyrus and left medial superior frontal gyrus, left middle temporal gyrus, right precentral gyrus, left precuneus after TSD. The middle temporal gyrus is involved in language processing, motion observing, and deductive reasoning (Xu et al., 2015). Our results indicated the changes in zFC values in the left middle temporal gyrus were positively correlated with the changes in scores of NCT-A, which reflects attention, and psychomotor speed. Abnormal FC with the left middle temporal gyrus may have impacted motor observation and deductive reasoning, subsequently affecting performance on the NCT-A. The left precuneus is a part of DMN, which participates in memory, visual and auditory attention, motor activity, and language processing (Raichle et al., 2001; Buckner et al., 2008). Precuneus is central in some highly integrated tasks, including visuospatial imagery, episodic memory retrieval, and self-processing operations (Cavanna and Trimble, 2006). The change in zFC with the left precuneus were negatively correlated with the change in scores of DST. DST requires participants to match symbols with corresponding numbers. Since language is a symbolic system, an individual’s language ability can also affect his ability to convert between different symbols to a certain extent (Hauser et al., 2002). Abnormal FC with the Left precuneus may have impacted language processing, subsequently affecting performance on the DST. The medial superior frontal gyrus is a part of the supplementary motor area (SMA). The precentral gyrus is where the primary motor cortex is located (Zhou et al., 2020). Our results suggested that after TSD, there were not only alterations in the spontaneous activity of some of the important brain regions but also an increase in FC between these regions and other areas of the brain, which may lead to alterations in associated cognitive functions and consequently affect the performance of neuropsychological tests.

Additionally, these altered mfALFF and zReHo value may reflect TSD-induced local neuronal injuries (Luo et al., 2016), and enhanced FC with other regions of the brain may represent a compensatory mechanism for such local injuries (Sheth et al., 2021; Hashimoto et al., 2023).

Our results demonstrated good diagnostic accuracy for the combined model in distinguishing between RW and TSD states. When each altered rs-fMRI feature was examined separately, the AUC values of each brain region with mfALFF changes were above 0.7, suggesting that the changes in mfALFF could be observed in most subjects. Combining multiple brain regions with mfALFF changes further improved diagnostic efficacy, approaching 0.8, thus indicating that simultaneous observation of these three brain regions in subjects could better distinguish whether they were in a sleep-deprived state. Compared to mfALFF, the diagnostic efficacy of zReHo changes in each brain region was slightly weaker, with only the right superior temporal gyrus exhibiting an AUC value greater than 0.7. Combining the zReHo changes in these brain regions did not improve the diagnostic efficacy, indicating that simultaneous observation of zReHo changes in multiple brain regions did not have a better diagnostic efficacy than observation of zReHo changes in individual brain regions. zReHo changes were found to have lower diagnostic power than mfALFF in distinguishing brain states. Besides, combining the FC changes in the left medial superior frontal gyrus and left precuneus also improved diagnostic efficacy. This indicates that FC changes in these regions are particularly sensitive to changes in brain states. Furthermore, combining all altered rs-fMRI features together, the AUC increased to 0.870. The ineffective rs-fMRI features were gradually eliminated, those with a major role were selected, and mfALFF of the bilateral postcentral gyrus and zFC of the left medial superior frontal gyrus and left precuneus were eventually identified. The DeLong’s test showed that the combined model had significantly better diagnostic efficacy than all single-parameter models, suggesting that these four rs-fMRI features exhibited significant changes in response to TSD and can potentially serve as imaging biomarkers for distinguishing between TSD and RW brain states. Future studies should focus further on mfALFF and FC changes in these regions.

The formulation of response strategies for medical staff under TSD conditions can be based on the discussions above. Firstly, when there are options for selecting treatment methods, it is best for medical staff under TSD conditions to avoid clinical procedures that require precise operations. Also, such treatments should be scheduled later when conditions allow for greater precision. Secondly, we suggest medical staff use auxiliary equipment whenever possible to assist in diagnosis and treatment to avoid overlooking critical factors due to incomplete medical history collection.

This kind of research can hopefully provide a theoretical basis for the evaluation system of cognitive impairment, warn medical staff of the high risk of cognitive decline, and promote the development of cognitive protection strategies and treatment measures to better protect the safety of medical staff and the life and health of patients.

The central focus of this study is to investigate changes in brain function before and after TSD in clinical staff. Given that functional alterations often correlate with structural changes, future research can further explore whether TSD indeed induces structural alterations. For instance, techniques such as Diffusion Tensor Imaging (DTI) and higher-order Diffusion Kurtosis Imaging (DKI) can be utilized to delve into subtle modifications of white matter tracts. Additionally, Micro-structural level magnetic Field Correlation (MFC) may be employed to assess alterations in iron deposition within the brain (Zhou, 2017). Furthermore, Voxel-Mirrored Homotopic Connectivity (VMHC) can be applied to observe changes in functional connectivity between bilateral hemispheres (Wei et al., 2018). These multimodal approaches, combining structural and functional assessments, provide a comprehensive understanding of the effects of TSD on the brain. Moreover, longitudinal follow-up studies in both animal models and human participants (Zhou, 2017) are crucial to evaluate the recovery and neuroplasticity changes in brain structure and function following TSD. In this study, the simple binary logistic regression was applied to build models of rs-fMRI features to distinguish between TSD and RW states (Dreiseitl and Ohno-Machado, 2002). More comprehensive classification models can be employed in future study to further improve the performance.

This study has the following limitations. First, despite our best efforts to replicate real-world clinical scenarios in the study environment, numerous uncontrollable confounding factors persist. Subsequent research focusing on multiple sleep deprivation cycles in individual subjects may offer improved control over confounding variables. Second, while medical staff are under high workload and mental stress during routine clinical night shifts, the working conditions faced by each subject are still unique, making it difficult to maintain consistency in the workload assigned to each subject, unlike that in laboratory settings. Thirdly, the determination of participants’ activity states (awake or asleep) lacks support from objective data. In future studies, it may be beneficial to incorporate activity recorders, such as actigraphy, to objectively record and validate the states of participants. Finally, the neuropsychological tests applied in this study were relatively simple and did not resemble tasks in routine clinical work. Sociability, speed, and errors during work were not directly assessed in this study. To gain a more comprehensive understanding of the cognitive impairments caused by TSD, future studies should include more neuropsychological tests, such as the Profile of Mood States (emotional state), Trail Making Test (visual attention and task switching), Psychomotor Vigilance Test (sustained vigilant attention), and NIH Cognition Toolbox (attention, memory, executive function, language, and perception) (Kielstein and Bernstein, 2014; Fullagar et al., 2015; Grumbach et al., 2020; Li et al., 2023). Moreover, in future research, it is important to further investigate the variations in physiological parameters during TSD, such as participants’ fatigue (Kayser et al., 2022), and their associations with abnormal brain activity.

In conclusion, spontaneous brain activity alterations occurred after TSD under workload, which might be associated with the reduced performance of medical staff in neurocognitive tests. These altered rs-fMRI features might be potential imaging biomarkers for distinguishing between TSD and RW brain states.

## Data availability statement

The raw data supporting the conclusions of this article will be made available by the authors, without undue reservation.

## Ethics statement

The studies involving humans were approved by Ethics Committee of Chongqing Hospital of Traditional Chinese Medicine. The studies were conducted in accordance with the local legislation and institutional requirements. The participants provided their written informed consent to participate in this study.

## Author contributions

CP: Conceptualization, Data curation, Formal analysis, Funding acquisition, Investigation, Methodology, Project administration, Resources, Software, Supervision, Validation, Visualization, Writing – original draft, Writing – review & editing. DiG: Data curation, Investigation, Software, Visualization, Writing – original draft. LL: Data curation, Investigation, Project administration, Resources, Writing – original draft. DX: Data curation, Investigation, Project administration, Resources, Writing – original draft. LN: Methodology, Writing – review & editing. HL: Methodology, Writing – review & editing. DaG: Conceptualization, Methodology, Project administration, Supervision, Validation, Writing – review & editing. HY: Conceptualization, Methodology, Project administration, Supervision, Validation, Writing – review & editing.

## Footnotes

^1^ https://www.fil.ion.ucl.ac.uk/spm/software/spm12/
